# Associations between women’s bargaining power and the adoption of rust-resistant wheat varieties in Ethiopia

**DOI:** 10.1016/j.worlddev.2024.106567

**Published:** 2024-06

**Authors:** Michael Euler, Moti Jaleta, Hom Gartaula

**Affiliations:** a International Maize and Wheat Improvement Center (CIMMYT), ILRI Sholla Campus, P.O. Box 5689, Addis Ababa, Ethiopia; b CIMMYT, Ethiopia; c CIMMYT, India

**Keywords:** Intra-household decision-making, Gender, Technology adoption, Resistant variety, Wheat, Ethiopia

## Abstract

The dynamics in intra-household decision-making are often neglected in literature on the adoption of agricultural innovations. However, households’ farm management decisions are often made following negotiations between female and male farmers. These may differ in terms of individual bargaining power and personal preferences. A better understanding of the links between gender roles in household decision-making and the adoption of technologies is postulated to enhance the uptake of innovations in smallholder farming systems. In this study, we use survey data from 1,088 wheat-producing households in Ethiopia to analyze the links between women’s role in household decisions concerning crop production and the adoption and turnover rates of rust-resistant wheat varieties. We interviewed female and male respondents from the same households, but separately, which facilitated capturing individual perceptions and the intra-household dynamics in decision-making. To account for observed heterogeneity that may simultaneously determine the level of women’s agency and varietal adoption by households, we employed Inverse Probability-Weighted Regression Adjustment (IPWRA). A positive association was found between women’s role in decision-making concerning choice of wheat seed and household adoption of rust-resistant wheat varieties and wheat varietal turnover. Spouses may be in agreement or have different opinions regarding their decision-making roles. The disagreement scenario in which the wife claims to have a role in decision-making is associated with lower adoption rates of rust-resistant wheat varieties and less frequent testing of new varties in recent growing seasons, compared to a scenario where both spouses agree that wives do not have a role. We conclude that gender-disaggregated data and the examination of intra-household decision-making can offer novel and valuable insights for designing and implementing strategies to enhance the uptake of agricultural technologies among smallholders. The results emphasize the need to include complementary perspectives on the intra-household decision-making process.

## Highlights

• The study analyzes associations between women’s bargaining power and the adoption of agricultural technologies.
• We assess linkages between gender roles in household decision-making and the use and turnover of rust-resistant wheat varieties in Ethiopia.
• We find a positive association between women’s agency and the adoption and turnover of rust-resistant wheat varieties and variety turnover.
• Spousal disagreement when the woman claims to have a role in decision-making tends to yield worse outcomes than spousal agreement.
• Examination of intra-household decision-making could enhance the uptake of agricultural technologies among smallholders.

## 1 Introduction

Household decisions often result from negotiations between wife and husband. While the literature offers different models to conceptualize the intra-household decision-making process, empirical evidence suggests that women’s empowerment has positive effects on a range of outcomes (Doss, 2013). These include household dietary diversity (Sariyev et al., 2020, Sraboni et al., 2014), agricultural production diversity (Sariyev, Loos, & Khor, 2021), reproductive, maternal, neonatal, and child health (Becker et al., 2006, Pratley, 2016), children’s education (Hatlebakk & Gurung, 2016), and nutritional status (Carlson, Kordas, & Murray-Kolb, 2015). Malapit and Quisumbing (2015) suggest that the relationship between women’s empowerment and nutritional outcomes can be complex and context dependent, i.e., women’s empowerment in household credit decisions was found to positively correlate with women’s dietary diversity, but not with their body mass index.

A growing body of literature also focuses on the linkages between the level of agreement of wife and husband in dual-headed households and health and welfare measures. For instance, in Nepal, Allendorf (2007) find that maternal and child healthcare outcomes improve when spouses agree that the wife is the main decision-maker. Based on national household data from Bangladesh, Story and Burgard (2012) show that the use of antenatal services and skilled delivery care improve when couples agree that decision-making is joint, and worsen when both agree that the husband is solely responsible or when the couple are in disagreement with each other. Ambler, Doss, Kieran, and Passarelli (2021) find that measures of Bangladeshi women’s well-being across health, employment, and participation in outside programs improve significantly when both spouses agreed that the wife is involved in decision-making. Based on data from 23 sub-Saharan countries, Annan, Donald, Goldstein, Gonzalez Martinez, and Koolwal (2021) show that well-being outcomes for women and children are high in households where the woman’s agency is recognized by her husband. The study also shows that when women take power — i.e., assign more decision-making power to themselves than their husbands do — households have better reproductive health and better children’s health outcomes. Donald, Doss, Goldstein, and Gupta (2023) find joint decision-making to be associated with lower levels of intimate partner violence and emotional and sexual violence among 31,243 couples from 12 African countries. Despite these well-documented welfare implications of intra-household decision-making dynamics, most household surveys focus on male household heads as the main source of information, potentially reducing the chance of adequately capturing the intra-household decision-making process (Acosta et al., 2020, Anderson et al., 2017).

The level of the wife’s bargaining power and of the husband’s agreement to her bargaining power may also influence the uptake of agricultural technologies. If empowered, women farmers may advocate for the adoption of agricultural technologies if these enhance individual or overall household well-being. Although extension and advisory services that incorporate female farmers can benefit households’ agricultural performance (Azzarri & Nico, 2022), extension systems often do not perceive women as training targets, thereby focusing on male farmers, and largely ignoring the preferences of female household members (Mudege, Mdege, Abidin, & Bhatasara, 2017). Although the adoption of agricultural technologies has received considerable attention in the literature (e.g., Schulz & Börner, 2023), only a small share of technology adoption studies explicitly include gender dynamics in intra-household decision-making in analytical frameworks (Fisher and Carr, 2015, Haider et al., 2018, Shibata et al., 2020). Among these, Shibata et al. (2020) show that there are gender disparities in decision-making about innovation uptake and benefits in Uganda, whereas Fisher and Carr (2015) find that differences in access to land, information, and credit shape women and men farmers’ adoption of drought-tolerant maize in Uganda. Haider et al. (2018) show that fertilizer adoption patterns differ between individually and collectively (female and male) managed plots in Burkina Faso.

A better understanding of the links between gender dynamics in household decision-making and related adoption decisions can enhance the efficiency and effectiveness of public extension systems, the uptake rates of modern innovations, agricultural productivity, and livelihoods in smallholder agriculture. In this study, we use survey data collected from Ethiopian wheat farmers to analyze how women’s role in wheat production decisions influences adoption dynamics and the turnover rates of rust-resistant wheat varieties. Different wheat rusts (stem, yellow, and leaf rust) pose a significant threat of causing devastating epidemics in Ethiopa (Jaleta, Hodson, Abeyo, Yirga, & Erenstein, 2019). To identify the factors affecting the adoption and turnover of rust-resistant wheat varieties in rural Ethiopia, we analyzed data collected from 1,088 wheat-producing households. In dual-headed households, both wife and husband were interviewed individually. We postulate that the adoption of newly released resistant varieties is positively associated with women’s role in decision-making and spousal agreement thereon. Our assumptions are based on two main expectations. First, building on empirical evidence suggesting that women farmers tend to be more risk averse (Ahmad et al., 2019, Jin et al., 2017), we hypothesize that men farmers are more eager to adopt high yielding varieties and more willing to mitigate rust pressure by application of fungicides within the season. We further hypothesize that due to their greater risk aversion, women farmers advocate for the adoption of rust resistant varieties to avoid liquidity problems that may arise from fungicide purchases in the growing season, which based on our survey data accounted for around 19 % of the average input costs in the 2021 wheat growing season. Second, it has been shown that intra-household communication and shared negotiation strategies lead to better health outcomes (Mullany, Hindin, & Becker, 2005). In the context of the adoption of agricultural technologies, women’s engagement in decision-making is expected to enhance the household’s exposure to relevant information on the benefits of the technology mainly due to spouses’ complementary social and institutional networks. Women’s participation in decision-making may also stimulate spousal discussion and reflection on potential implications of the varietal choice decision. The use of gender-disaggregated data allows us to capture and contrast different perceptions on intra-household decision-making, as well as potentially diverging perspectives on varietal choice and seed acquisition channels.

The rest of the paper is structured as follows. The next section briefly introduces the Ethiopian wheat sector and the relevance of rust-resistant varieties. Section 3 presents the empirical strategy, and Section 4 the underlying dataset. Section 5 contains the estimation results and a discussion of the findings. Section 6 concludes.

## 2 Wheat production and rust epidemics in Ethiopia

Ethiopia is the second-largest wheat producer in Africa, with an aggregate grain production of 5.5 million tons (Food and Agriculture Organization of the United Nations, 2022) and 4–5 million farmers engaged in cultivation (Central Statistics Agency, 2018). Since 2000, the country’s wheat area has increased by over 70 %, making it the third most cultivated crop after teff and maize (FAO, 2022). However, wheat yield in the country (2.67 metric tons per hectare in 2021) is significantly below the global average (3.49 metric tons) (FAO, 2022). The Ethiopian Highlands have long been known as a hot spot for wheat rust (Saari & Prescott, 1985). Stem rust and stripe rust are major biotic constraints to wheat production in the country (Meyer et al., 2021). With recurrent epidemics in the last decade, the emergence of new strains of wheat rust exacerbated production risks. During the wheat stripe rust epidemic of 2010 (Olivera et al., 2015) and the stem rust outbreak in 2013 (Hodson et al., 2020), the average loss amounted to about 50 % of the national crop yield, and in some regions the total production was lost (Olivera et al., 2015).

The breeding and diffusion of resistant wheat germplasm form a crucial component of the research-and-development (R&D) strategy against the risk of rust outbreaks (Jaleta et al., 2019, Meyer et al., 2021). Two important *ex ante* mitigation strategies to curtail the risk of rust outbreaks in smallholder systems include expanding the area under resistant varieties and increasing the genetic diversity of wheat varieties (Jaleta et al., 2019). Recent evidence based on DNA fingerprinting shows that rust-resistant bread wheat varieties released since 2010 have been widely adopted by smallholder farmers across Ethiopia (Hodson et al., 2020, Jaleta et al., 2019). Farmers seem to be responsive to the management of rust diseases, as evidenced by a large-scale disadoption of susceptible varieties and replacement with newer, rust-resistant varieties after the 2010/11 epidemic (Jaleta et al., 2019).

## 3 Empirical strategy

### 3.1 Measuring women’s bargaining power

Our analysis focuses on the links between women’s bargaining power and the adoption of new and resistant wheat varieties (released after the 2010/11 rust epidemic) and wheat varietal turnover in Ethiopia. To do so, we identify and estimate the determinants of women’s intra-household bargaining power in wheat production decisions. As bargaining power cannot be observed directly, we rely on proxy variables. The literature suggests several ways to indirectly capture women’s bargaining power in household decision-making, including the share of income earned by women, employment outside the home, control over and ownership of household assets, influence in household decision-making, etc. (Doss, 2013). Since our prime interest is to capture the level of women’s bargaining power in the context of crop production and wheat varietal choice, we rely on two proxy variables: i) the role of the wife in household decisions concerning varietal choice in wheat production; and ii) the level of agreement between wife and husband in terms of women’s engagement in household decisions on wheat varietal choice.

The outcomes concerning women’s role in household crop production decisions are constructed based on interviews conducted individually with wife and husband from the same household. We have two sets of observations for each outcome: decision-making agency within the household according to the wife and husband. Both respondents were asked separately to assign themselves to one of three decision categories describing their individual role in household decisions on wheat variety choice over the past 12 months. The responses were categorical: “decisions taken independently”, “joint decisions”, and “not involved in decision-making”. Comparing the responses of wife and husband within a household, the level of intra-household spousal agreement variable was developed as follows: “agreement that the wife has no role”; “agreement that the wife has a role”; “disagreement: the husband claims that the wife has a role, but the wife denies this”; and “disagreement: the wife claims that she has a role, but the husband denies this”.

These four agreement/disagreement categories not only capture the recognition of women’s power (“agreement on the wife's role”) but also distinguish between households in which female farmers are “power-takers” (the wife claims that she has a role, but the husband denies this) and households in which male farmers are “power-givers” (the husband claims that the wife has a role, but the wife denies this). These two disagreement categories enable a more nuanced understanding of women’s empowerment (Annan et al., 2021) because empowered individuals may both recognize and claim their rights (O'Hara & Clement, 2018).

Given the categorical nature of the established proxy variables for women’s bargaining power, we used binominal and multinominal logistic regression models to identify their determinants and estimate the strength of their influence. The commonly used explanatory variables found in the literature are women’s education, age, asset holding, and income, and the difference in these variables compared to those of her husband (Annan et al., 2021, Doss, 2013). Together with the characteristics of the household members, general household features, the household’s engagement in personal and institutional networks, and location dummies are used as regressors.

### 3.2 Modeling associations of women’s bargaining power and technology adoption

In the next step, we modeled the influence of women’s bargaining power proxies on households’ adoption of varieties released after 2010 and varietal turnover. As indicated in Section 2, Ethiopia experienced several wheat rust epidemics in recent years, including the yellow rust epidemic in 2010 (Jaleta et al., 2019). A crucial coping strategy to deal with constant rust pressure is rapid varietal turnover and constant replacement of older germplasm. Several resistant varieties have been released since 2010 (Jaleta et al., 2019). Against this background, we estimated the effect of women’s bargaining power proxies on the following: i) households’ adoption status (binary variable) in the 2021 growing season of wheat varieties released after 2010; ii) the average age of the wheat varieties grown on the farm (in years, as of 2021); and iii) whether the household introduced/tried any new wheat varieties during the main growing seasons between 2015 and 2021 (binary variable). Relying on the terminology of treatment effects estimation, we considered women farmers’ influence on households’ decisions on wheat varietal choice as the treatment, and estimated the average treatment effect on the treated (ATT), which is the average difference in outcomes of households with and without active participation by women farmers in decisions about wheat varietal choices (Takahashi and Barrett, 2014):

(1) $$\mathrm{ATT}\equiv E\left\{Y_{\mathrm{iP}}-Y_{\mathrm{iN}}|T_{i}=1\right\}\;=E\left(Y_{\mathrm{iP}}\right|T=1)-E\left(Y_{\mathrm{iN}}\right|T_{i}=1\}$$

With E {.} being the expectation operator, the potential outcomes for households with $(Y_{\mathrm{iP}}$) and without $\left(Y_{\mathrm{iN}}\right)$ women farmers’ participation in decisions on wheat varietal choice, and $T_{i}$ the treatment indicator equal to 1 if women farmers participate in decision making and 0 otherwise. $E\left(Y_{\mathrm{iN}}\right|T_{i}=1\}$ — the outcome of households with womens’ participation in decision-making had women not participated in decision-making — cannot be observed. Using outcomes of households without womens’ participation on decision-making $E\left(Y_{\mathrm{iN}}\right|T_{i}=0\}$ instead of unobserved counterfactuals can introduce a bias in ATT estimates (Takahashi & Barrett, 2014), especially if covariates of the treatment and outcome equations are correlated. We use the Inverse Probability-Weighted Regression Adjustment (IPWRA) proposed by Wooldridge (2010), which has been employed in a range of studies that assess the impacts of agricultural technology adoption with observational data (e.g., Wossen et al., 2017). The IPWRA estimator is double-robust, as it produces consistent results if either the treatment or the outcome equations are misspecified (Wooldridge, 2010), and accounts for observed heterogeneity that may simultaneously determine the level of women’s role in decision-making, spousal agreement thereon, and the set of outcome variables. After Imbens and Wooldridge (2009), the ATT in the IPWRA model is estimated in two steps. Assuming the linear outcome equation

(2) $$Y_{i}=\alpha_{i}+\beta_{i}x_{i}+\varepsilon_{i}$$

for i = [0 1], propensity scores are modelled as e^(xi)=px;γ^, after which linear regression is used to estimate $\left(\alpha_{0},\beta_{0}\right)$ and $\left(\alpha_{1},\beta_{1}\right)$ using inverse probability-weighted least squares. Formally:

(3) $$\underset{\alpha_{0},\beta_{0}}{\min}\;\overset{N}{\underset{i=1}{\sum }}\frac{{\left(y_{i}-\alpha_{0}-\beta_{0}x_{i}\right)}^{2}}{p\left(x_{i};\hat{\gamma }\right)}\;if\;T_{i}=0\;$$

(4) $$\underset{\alpha_{1},\beta_{1}\;}{\min}\overset{N}{\underset{i=1}{\sum }}\;\frac{\;{\left(y_{i}-\alpha_{1}-\beta_{1}x_{i}\right)}^{2}}{\left(1-p\left(x_{i};\hat{\gamma }\right)\right)}\;if\;T_{i}=1$$

The ATT is the difference between Eq(4) and Eq(3).

(5) ATT=1Np∑i=1Np[α^1-β^1xi-(α^0-β^0xi)]

With α^1,β^1 being the estimated inverse probability-weighted parameters for treated households, i.e., households with participation by women in wheat varietal choice decisions, and α^0,β^0 the estimated inverse probability-weighted parameters for untreated households, i.e., households with no particpation by women in wheat varietal choice decisions and Np the number of observations with women's participation in decision-making.

The IPWRA relies on three assumptions. The first is the conditional-independence (CI) assumption, which states that potential outcomes are not correlated with treatment once all observable variables are controlled for. In other words, no variables should influence the treatment and outcome equations simultaneously. Including a wide range of control variables in $x_{i}$ is a strategy that minimizes the chances of violation of the CI assumption (Wooldridge, 2010). In this study, the covariates included in the models represent a diverse set of individual, household, and context variables that are expected to influence women farmers’ participation in decision-making. The selection of covariates is guided by empirical work on the correlates of individual bargaining power in household decision-making (e.g., Annan et al., 2021, Johnson et al., 2016, Mishra and Sam, 2016). The second assumption is the overlap assumption that stipulates that each individual has a positive probability of receiving treatment. This assumption holds when for each household with womens’ participation in decision-making in the sample, we observe households without womens’ participation in decision-making with similar covariates (Manda et al., 2018). The third assumption is the independent and identically distributed (i.i.d.) sampling assumption. The i.i.d. ensures that the outcome and treatment status of a household are independent of the outcome and treatment status of other households in the population.

While IPWRA accounts for observed heterogeneity, we cannot rule out the existence of unobserved confounders that may simultaneously influence the level of women’s bargaining power in the household and the decision to adopt rust-resistant varieties. Unobserved factors include individual norms, concepts, and beliefs regarding gender roles in household decision-making. The use of instrumental variables (IV) is one strategy to establish causality. Due to the lack of a relevant, exclusive and unconfounded IV, we rely on IPWRA estimates and avoid the use of causal language in interpreting the results. As robustness check, we also estimated treatment effects using propensity score matching, which yielded very similar results compared to IPWRA estimates.

## 4 Data

To assess the links between intra-household gender inequalities and the uptake of innovations, we designed a survey to interview the wife and husband from the same household separately, allowing us to record and contrast perceptions on intra-household decision-making, wheat variety choice, and varietal turnover. Differences in intra-household decision-making regarding farm activities were elicited through questions on their role in decision-making regarding wheat production, variety choice, plot and seed selection, marketing, and allocation of seed for the forthcoming season. This approach allowed capturing the level of women involvement in household-level decisions on wheat varietal choice, as well as the level of agreement among the wife and husband on their roles within the household. The survey also included questions on socioeconomic household characteristics, use of inputs in wheat production, farmland endowments, access to markets and institutions, food consumption and food insecurity, etc.

The sampling framework of the survey was structured with the objective of obtaining a proportional distribution of observations across Ethiopia’s major wheat agroecological zones (Jaleta et al., 2019). The survey was conducted between July and September 2021 and constituted the third survey round with previous data collections in 2011 and 2014. We used the latest survey round in the analysis, as data on intra-household decision-making was only elicited in the 2021 interviews. As shown in Table 1, the 2021 survey covered households from Ethiopia’s major wheat-growing regions: Amhara, Oromia, and the Southern Nations Nationalities and Peoples (SNNP) Regional States. In the original sampling framework, a stepwise stratified random sampling was followed. First, wheat-growing districts were randomly selected from each region. Second, wheat-growing *kebeles*, the lowest administrative unit in Ethiopia, were randomly selected from each district. Finally, in each kebele, 14–18 randomly selected wheat-growing households were interviewed (Jaleta et al., 2019). The survey contained 9.6 % female-headed households (n = 104). In 86.8 % of the interviewed households, both female and male spouses were present (n = 944).

**Table 1:** Number of households included in the survey by region.

Region	Total		Female-headed households		Households with both spouses (conventionally male-headed)	
	N	% (of the column total)	N	% (of the region)	N	% (of the region)
SNNP	167	15.4	13	7.8	138	82.6
Amhara	243	22.3	20	8.2	215	88.5
Oromia	678	62.3	71	10.5	591	87.2
*Total*	*1088*	*100.0*	*104*	*9.6*	*944*	*86.8*

Notes: N stands for the number of observations. Missing observations (n = 40) are male-headed households without a wife.

## 5 Results and discussion

### 5.1 Women’s role in crop production decisions

Table 2 shows the role of women farmers in household decisions concerning wheat varietal choice over a period of 12 months, as perceived by the wife and husband. In general, decision-making is male-centric: according to wives’ perceptions, they are not involved in decision-making in 54 % of households. In about 39 % of households, they contribute to joint decision-making, and only in 8 % of households do wives make decisions independently. These values are slightly different when elicited from husbands. Most notably, a significantly larger share of men farmers indicate that the wife is the sole decision-maker in wheat varietal choice. This contrasts with observations made in the context of the uptake of agricultural innovations in other African countries. For instance, husbands over-proportionally claim the position of main decision-maker regarding the introduction of innovations compared to their wives in Uganda (Shibata et al., 2020). The differences may be related to the fact that the questionnaire administered for this study did not frame decision-making on wheat varieties around the introduction of innovations. Nonetheless, it is not unusual that women contribute more to production and management of wheat than men in certain locations of Ethiopia (Ogato, Boon, & Subramani, 2009).

**Table 2:** Women’s role in household decision-making in wheat varietal choice.

	Wife’s role in wheat varietal choice decisions, according to:			
		
	N	%	N	%
Wife makes decisions	71	7.5	145	15.4^***^
Both spouses jointly make decisions	365	38.7	328	34.7^***^
Wife not involved in decision-making	508	53.8	471	49.9^***^
*Total*	*944*	*100.0*	*944*	*100.0*

Notes: N stands for the number of observations, and % values are that of column totals. Wife’s role in wheat varietal choice decisions as perceived by wife () and husband (). ^***^ denotes that differences between responses are statistically significant at 1%, based on the Wilcoxon signed-rank test for equality of matched pairs.

In Table 3, we summarize the degree of agreement between wife and husband regarding women’s role in household decision-making on wheat varietal choice. Following an approach suggested by Annan et al. (2021), the answers can be grouped into four categories: agreement that the wife has no role in household wheat varietal choice decisions (scenario A); agreement that the wife has a role in decision-making (scenario B); disagreement: “power-giving”, with the husband assigning a greater role in decision-making to the wife than the wife assigns to herself (scenario C); disagreement “power-taking” with the wife assigning to herself a greater role in decision-making than the husband assigns to her (scenario D). While implications of scenarios (A) and (B) are straightforward, scenarios (C) and (D) differ in terms of intra-household bargaining power dynamics, as recognition and claim of power (scenario D) could be considered greater empowerment than the delegation of influence by someone else (scenario C) (O'Hara & Clement, 2018).

**Table 3:** Perceptions of women’s role in household decision-making by wife and husband.

	Agreement on wife’s role in wheat varietal choice	
	N	%
Agreement on no role for wife (A)	306	32.4
Agreement on a role for wife (B)	262	27.8
Disagreement, “power-giving” husband (C)	209	22.1
Disagreement, “power-taking” wife (D)	167	17.7
*Total*	*944*	*100.0*

Notes: N stands for the number of observations, and % values are that of column totals.

About 40 % of couples disagreed on the woman’s role in household wheat varietal choice decisions. Similar patterns have been reported by Annan et al. (2021), who find that spousal responses regarding decision-making on major household purchases systematically differ in 21 out of 23 sub-Saharan African countries. Ambler et al. (2021) report 39 % of spouses to disagree on their mutual role in agricultural production decisions in Bangladesh, with 33 % of disagreements stemming from the wife claiming power (scenario D). Disagreement over authority in household decisions between spouses has also been reported for production decisions made by rural households in Tanzania (Anderson et al., 2017), innovation uptake and benefits in Uganda (Shibata et al., 2020), and uptake of agricultural practices also in Uganda (Acosta et al., 2020).

### 5.2 Correlates of women’s role in wheat varietal choice and spousal agreement

Before assessing the implications of women’s bargaining power proxies on variety adoption and turnover, we assess the factors that contribute to a woman’s role in making decisions on wheat varietal choices and the level of agreement among spouses on this role. When analyzing correlates of women’s bargaining power proxies, previous studies have used individual attributes of wife and husband, such as education, employment status, earnings, asset ownership, and the differences between individual household members regarding these attributes, as well as common household characteristics (Anderson et al., 2017, Annan et al., 2021). We use a set of individual socio-demographic characteristics of the wife and their relative expressions in relation to the husband, as well as overall household features, personal and institutional networks, information access variables, and location dummies to model the level of women’s role in household wheat varietal choices and of spousal agreement on this role.

We first model correlates of a woman’s role in making decisions about the household’s wheat varietal choices. Due to the unbalanced distribution of observations across decision-making categories — as only 7.5 and 15.4 % of observations fall into the “wife makes the decision” category in the wife and husbands interviews, respectively — a binary logit model is estimated. The dependent variable of the model is binary and takes on the value 1 if the wife is the main decision maker or if the decision is joint, and 0 otherwise. Table 4 shows the marginal effects for selected covariates. Marginal effects indicate how the probability of observing the outcome variable changes when the covariate changes by one unit. Since participation in decision-making is subjective to wives' and husbands' perceptions, we estimate two models. This approach allows us to contrast the spouses’ perceptions. The full model results are provided in the supplementary material (Table S1).

**Table 4:** Correlates of women’s role in household decisions on wheat varietal choice.

	Wife engages in decisions on wheat varietal choice *(dummy)*	
	(1)	(2)
	As per female interview	As per male interview
Use rights of agricultural land		
Size of land with wife’s or joint use rights is equal to size of land with husband’s use rights (*dummy) ^#^*	−0.05 (0.09)	0.34^***^ (0.09)
Size of land with wife’s or joint use rights is larger than size of land with husband's land use rights *(dummy) ^#^*	0.27^***^ (0.03)	0.20^***^ (0.03)
Ownership of non-agricultural items		
Wife’s or joint ownership of non-agricultural items is equal to husband's ownership of non-agricultural items (*dummy) ^#^*	0.09^***^ (0.03)	0.09^***^ (0.03)
Wife’s or joint ownership of non-agricultural items is larger than husband's ownership of non-agricultural items *(dummy) ^#^*	0.14^***^ (0.06)	0.02 (0.06)
Ownership of agricultural items		
Wife’s or joint ownership of agricultural items is equal to husband’s ownership of agricultural items (*dummy) ^#^*	0.07^**^ (0.03)	0.05 (0.03)
Wife’s or joint ownership of agricultural items is larger than husband's ownership of agricultural items *(dummy) ^#^*	0.05 (0.08)	0.12 (0.08)
N	944	944
Pseudo R^2^	0.15	0.14
Log likelihood	−553.70	−560.48
chi^2^	146.64	163.12

Notes: Results are based on binary logit models. The dependent variable takes on the value 1 if the wife is the main decision maker in household decisions regarding wheat varietal choice, or if the decision is joint, and 0 otherwise. Standard errors in parentheses are robust. Estimates are marginal effects indicating the change in probabilities of observing the outcome variable when the independent variable increases by one unit. * p < 0.1, ^**^ p < 0.05, ^***^ p < 0.01. ^#^ The reference category is lower wife's or joint ownership of the respective variable in relation to husband’s sole ownership. Only selected covariates are shown. Full model results are contained in Table S1.

The results indicate that formalized agricultural land use rights are strongly associated with a woman’s active role in making household decisions on wheat varietal choices. Wife’s or joint use rights of an equal (model 2), or larger (models 1 and 2) area of agricultural land in relation to husband’s sole use rights increases the likelihood of a woman’s engagement in making household decisons on wheat varietal choice. In Ethiopia, access to agricultural land is only by government allocation, inheritance, gift, and land leasing. As such, land cannot be sold, exchanged, or mortgaged (Bodurtha et al. 2011, cited in Leta, Berlie, & Ferede, 2021), which makes it a scarce resource and potential lever in intra-household bargaining process. However, both men and women farmers can obtain formalized rights to land, including the right to use land for agricultural purposes, lease out, and bequeath land to family members (Tura, 2018).

Women’s or joint ownership of agricultural and non-agricultural household items in relation to exclusive men’s ownership enhances the chances of participation in wheat varietal choice. Notably, the associations are stronger for model specifications based on wives' perceptions. Previous studies on intra-household decision-making have identified control over and ownership of household assets as significant determinants of bargaining power (Annan et al., 2021, Johnson et al., 2016, Mishra and Sam, 2016, Shibata et al., 2020).

As shown in Table 2, perceptions of the women’s role in household decision-making can differ between wife and husband. Table 5 presents correlates of spousal agreement on the women’s role in household crop production decisions. Data points are relative risk ratios for selected covariates based on multinominal logit models, indicating the change in relative risks of observing the outcome variable as the independent variable increases by one unit. Full model results are contained in the Supplementary Materials (Table S2).

**Table 5:** Correlates of agreement on women’s role in household decisions on wheat varietal choice decisions.

	Spousal agreement on wife’s role in wheat varietal choice		
	Agreement on wife’s role (B)	Disagreement, husband assigns greater role to wife (C)	Disagreement, wife assigns herself greater role (D)
Use rights of agricultural land			
Size of land with wife’s or joint use rights is equal to size of land with husband's land use rights (*dummy) ^#^*	4.20^**^	3.92^***^	0.00^***^
	(2.82)	(1.93)	(0.00)
Size of land with wife’s or joint use rights is larger than size of land with husband's land use rights *(dummy) ^#^*	7.15^***^	1.87^***^	2.69^***^
	(1.81)	(0.39)	(0.67)
Ownership of non-agricultural items			
Wife’s or joint ownership of non– agricultural items is equal to husband’s ownership of non-agricultural items (*dummy) ^#^*	2.07^***^	2.07^***^	2.24^***^
	(0.44)	(0.43)	(0.55)
Wife’s or joint ownership of non-agricultural items is larger than husband’s ownership of non-agricultural items *(dummy) ^#^*	2.09*	0.85	2.20*
	(0.85)	(0.39)	(0.91)
Ownership of agricultural items			
Wife’s or joint ownership of agricultural items is equal to husband’s ownership of agricultural items (*dummy) ^#^*	1.71^**^	1.01	1.08
	(0.37)	(0.21)	(0.25)
Wife’s or joint ownership of agricultural items is larger than husband’s ownership of agricultural items *(dummy) ^#^*	2.28	1.84	1.34
	(1.16)	(0.98)	(0.71)
N	944 0.17 −1063.99 441.90		
Pseudo R2			
Log likelihood			
Chi2			

Notes: Results are based on multinominal logit models. Dependent variable is the level of agreement on the wife’s role in household decisions on wheat varietal choice. Standard errors in parentheses are robust. Estimates indicate the relative risk ratios of observing the respective level of the outcome variable relative to observing the base level (agreement on no role for female spouse in wheat varietal choice (scenario A)) when the independent variable increases by one unit. * p < 0.1, ^**^ p < 0.05, ^***^ p < 0.01. ^#^ The reference category is lower wife’s or joint ownership of the respective variable in relation to husband’s sole ownership.

The results show that women’s or joint use rights of agricultural land, ownership of non-agricultural and agricultural items are strongly associated with agreement scenario B (both spouses acknowledge women’s role in decision-making), or with scenarios in which either the male spouse (scenario C) or the female spouse (scenario D) claim an active role by the woman in decision-making. Notably, wives' or joint ownership of agricultural land increases the likelihood of spousal agreement on an active role by the woman, the likelihood of the husband assigning a role to the wife even if she claims not to have one (scenario C), as well as the likelihood of wives claimng an active role in decision-making (Scenario D). Annan et al. (2021) also find that women’s ownership of land is associated with women claiming power in household’s decisions on large purchases.

### 5.3 Associations between women’s role in varietal choice decisions and adoption and turnover of wheat varieties

Is the woman’s role in household decisions on wheat varietal choice and spousal agreement thereon associated with the adoption of rust-resistant wheat varieties and the age of the varieties in farmers’ plots? Before presenting the estimation results, we summarize the set of outcome variables by the level of women’s role in decisions on wheat varietal choice in Table 6. The level of the woman’s role is based either on the interview with the wife or the husband. Outcome variables are (1) whether the household is cultivating a wheat variety with rust-resistant traits released after 2010 (dummy); (2) the average age of the wheat varieties grown by the household across all wheat plots in the 2021 cropping season (years); and (3) whether the household has tried out new wheat varieties between 2015 and 2021 (dummy). Outcome variables are also computed based on information reported by either the wife or the husband.

**Table 6:** Summary statistics for the adoption and turnover of wheat varieties by the level of women’s role in household decisions on wheat varietal choice.

	Wife makes decision (I)		Both spouses make decision (II)		Wife not involved in decision (III)		Total		I = III		II = III	
												
	(n = 71)	(n = 145)	(n = 365)	(n = 329)	(n = 508)	(n = 470)	(N = 944) ^#^					
Household grows wheat variety released after 2010 *(dummy)*	0.63	0.59	0.59	0.62	0.50	0.50	0.54	0.56^+^	***	***	***	***
	(0.49)	(0.49)	(0.49)	(0.49)	(0.50)	(0.50)	(0.50)	(0.50)				
Average age of wheat varieties grown by household^##^	8.39	14.02^+++^	11.69	11.48	13.62	12.58^+++^	12.45	12.42	***	***	***	***
	(2.18)	(9.78)	(6.30)	(6.06)	(9.25)	(8.12)	(7.92)	(7.81)				
Household tested new varieties during 2015–21 *(dummy)*	0.13	0.58^+++^	0.37	0.50^+++^	0.42	0.45^+^	0.38	0.49^+++^	***	***	***	**
	(0.34)	(0.50)	(0.48)	(0.50)	(0.49)	(0.50)	(0.49)	(0.50)				

Notes: Decision-making role categories and outcome variables are either computed based on wifes () or husbands () interviews. ^***^, ^**^, * indicate significant differences of means between respective comparisons between columns I, II, and III by gender of respondent at the 1, 5 and 10 % level, respectively. NS is not significant. ^+++, +^ indicate significant differences of means between the wife's and husband's perceptions within each panel at the 1 and 10 % level, respectively. ^#^Total number of observations for average age of wheat varieties is N = 743 () and N = 771 (). ^##^ number of observations across role in decision-making categories is : wife makes decision n = 54, joint decision n = 304, no role n = 385; : wife decision maker n = 123, joint decision n = 272, no role n = 376. The difference in sample size is due to cultivation of varieties that could not be matched with varieties from official register and no release date could be determined.

The means of outcome variables are compared between the scenario in which the wife is the main decision-maker and the scenario in which the wife has no role in decision making (in column ‘I = III’), and between the scenario of joint spousal decision-making and the scenario of no role for the wife in decision-making (column ‘II = III’). Comparisons of scenarios are based on the information collected from either the wife or the husband in constructing the outcome variables and the levels of the woman’s role in decision-making.

Results show a more frequent adoption of varieties released after 2010, as well as faster turnover of wheat varieties when the wife has an active role in decision-making compared to the wife having no role in decision-making. Differences are observed when the wife is either the main decision maker (column I) or when decisions are made jointly (column II). We find results to be sensitive to whether wives’ or husbands’ interviews are considered. For instance, when based on husbands’ interviews, the oldest wheat varieties are used by households where the wife is the main decision-maker (column I). Likewise, husbands indicate that a significantly larger share of households has tested new varieties between 2015 and 2021 when the wife has a role in household wheat varietal choice decisions. When, in turn, based on the wives’ information, households are found to test new varieties significantly less frequently when women are the main decision-makers (column I).

Does the level of women’s role in wheat varietal choice affect wheat adoption decisions and varietal turnover? The descriptive results in Table 6 do not control for the presence of potential confounding factors which may influence observed group means of outcome variables. Table 7 shows the results of IPWRA models for the set of outcome variables. Outcome variables and treatments are generated based on the information of either the wife or the husband. Accordingly, we have two observations for outcome variables and treatments for each household. Table 7 shows the average treatment effects on the treated (ATT) of the perceived role of the wife in household wheat varietal choice. All the models contain additional covariates, including characteristics of individual household members, household and farm features, agricultural asset holdings, and information access dummies. The full list of covariates contained in the models is included in the supplementary materials (Table S3). One assumption of IPWRA is that each individual has a positive probability of receiving treatment. In the present case, women’s participation in wheat varietal choice must have a positive probability across the distribution of covariates included in the models. Corresponding tests for deployed IPWRA did not reject the hypothesis of balanced covariates. Graphical representations of estimated densities of probabilities of treatments are contained in the supplementary materials (Figures S1 and S2).

**Table 7:** Associations between women’s role in decisions on wheat varietal choice and outcome variables.

	Household adopted wheat variety released after 2010 (dummy) Based on wife's or husband's interview:		Average age of grown wheat varieties (years) Based on wife's or husband's interview:		Household has tested new varieties between 2015 and 2021 (dummy) Based on wife's or husband's interview:	
	(1)	(2)	(3)	(4)	(5)	(6)
						
**Average treatment effect on the treated (ATT)**						
Wife has a role *vs. wife has no role*	0.08^**^ (0.04)	0.07* (0.04)	−1.67^***^ (0.67)	−0.04 (0.61)	−0.09^***^ (0.04)	0.13^***^ (0.04)
**Potential-outcome means (POmeans)**						
Wife has a role	0.51^***^ (0.03)	0.54^***^ (0.03)	12.87^***^ (0.58)	12.23^***^ (0.50)	0.43^***^(0.03)	0.39^***^ (0.03)
*Observations*	*944*	*944*	*743^#^*	*771^#^*	*944*	*944*

Notes: Treatment and outcome variables are based on  (models 1,3,5) or  (models 2, 4,6) interviews. ATT are average treatment effects on the treated. Robust standard errors in parentheses. ^#^Differences in sample size are due to farmers identifying a variety which had no match in the variety register, and therefore for which no varietal age could be calculated. * p < 0.10, ** p < 0.05, *** p < 0.001. ^#^Total number of observations for average age of wheat varieties is N = 743 () and N = 771 (). Additional covariates include characteristics of wife and husband, household and farm characteristics, information access proxies, and location dummies. The full list of covariates and probability density plots are contained in supplementary materials (Table S3 and Figures S1 and S2).

Estimation results suggest that women’s engagement in wheat varietal choice is associated with a higher likelihood of growing rust-resistant and younger wheat varieties. However, results are sensitive to who in the household responds (wife or husband) in two out of three outcome variables. For instance, results based on wives’ interviews show that women’s role in decision-making is associated with significantly younger wheat varieties (model 3), which is not observed in the model using information from the husbands (model 4). Similarly, the testing of new varieties is more likely when male farmers perceive the wife to have a role in crop production decisions (model 6), and less likely when models are based on wives’ interviews (model 5). While we cannot assess whether results based on wife or husband interviews more accurately reflect real-life conditions, the findings underline potential shortcomings when including only one perspective on the intra-household decision-making process.

### 5.4 Associations between spousal agreement on women’s role in crop farming decisions and adoption and turnover of wheat varieties

We have seen that differences in the perception of the woman’s role in household crop production decisions between wife and husband can be significant. Several empirical studies have shown associations between spousal agreement on the woman’s role in decision-making and socioeconomic welfare indicators (Allendorf, 2007, Ambler et al., 2021, Annan et al., 2021, Story and Burgard, 2012). In the context of the adoption of rust-resistant wheat varieties, spousal agreement on the woman’s role in wheat varietal choice decisions may affect varietal adoption and turnover. Table 8 presents means of outcome variables by level of spousal agreement on the woman’s role in decisions on wheat varietal choice. Corresponding tests of differences of means of outcome variables by level of spousal agreement are shown in Table 9.

**Table 8:** Summary statistics for the adoption and turnover of wheat varieties by level of spousal agreement on the women’s role in household decisions on wheat varietal choice.

	Agreement on female spouse's role in wheat varietal choice										
	Agreement on no role (Scenario A) (n = 306)		Agreement on role (Scenario B) (n = 262)		“Power-giving” (Scenario C) (n = 209)		“Power-taking” (Scenario D) (n = 167)			Total (N = 944) ^#^	
											
Household grows wheat variety released after 2010 *(dummy)*	0.48 (0.50)	0.48 (0.50)	0.66 (0.47)	0.66 (0.47)	0.52 (0.50)	0.56^**^ (0.50)	0.50 (0.50)	0.56^**^ (0.50)		0.54 (0.50)	0.56* (0.50)
Average age of wheat varieties grown by household *(years)^##^*	13.68 (9.33)	13.62^**^ (9.10)	11.71 (6.41)	11.48 (56.00)	13.43 (9.00)	13.35 (9.04)	10.34 (5.16)	10.77 (5.65)		12.45 (7.92)	12.42 (7.81)

Household tested new varieties from 2015 to 2021 *(dummy)*	0.41 (0.49)	0.54^***^ (0.50)	0.40 (0.49)	0.50^***^ (0.66)	0.44 (0.50)	0.55^***^ (0.50)	0.22 (0.42)	0.31^***^ (0.46)		0.38 (0.49)	0.49^***^ (0.50)

Notes: Agreement categories are: “agreement on no role for wife” (scenario A); “agreement on wife’s role” (scenario B); “the husband says that the wife has a role and the wife disagrees” (scenario C); and “the wife says that she has a role and the husband disagrees” (scenario D). ^#^Total number of observations for average age of wheat varieties is N = 743 (female interviews) and 771 (male interviews). Observations for average age of wheat varieties according to scenarios are for female interviews scenario A: n = 229, scenario B: n = 226, scenario C: n = 160, scenario D: n = 128; for male interviews: scenario A: n = 238, scenario B: n = 223, scenario C: n = 170, scenario D: n = 140. ^***^, ^**^, * indicate difference of mean values of outcome variables between female and male-based construction of respective outcome variable within agreement scenario at the 1, 5, and 10 % level respectively.

**Table 9:** Tests for significant differences of means of outcome variables by spousal agreement on women’s role in wheat varietal choice.

		Agreement on no role (A) = Agreement on role (B)	Agreement on no role (A) = “Power-giving” (C)	Agreement on no role (A) = “Power-taking” (D)	Agreement on role (B) = “Power-giving” (C)	Agreement on role (B) = “Power-taking” (D)	“Power-giving” (C) = “Power-taking” (D)
Household grows wheat variety released after 2010 *(dummy)*		***	*NS*	*NS*	***	***	*NS*
		***	**	**	***	***	*NS*
Average age of wheat varieties grown by household *(years)*		***	*NS*	***	***	***	***
		***	*NS*	***	***	*	***
Household tested new varieties during 2015–21 *(dummy)*		*NS*	*NS*	***	*NS*	***	***
		*	*NS*	***	*	***	***

Notes: ^***^, ^**^, * denote pairs of mean values of outcome variables are significantly different at the 1, 5, and 10% level, respectively. NS denotes ‘not significant’.

According to the descriptive results and related tests of equality of means, spousal agreement on a woman’s active role in variety selection (scenario B) is associated with more frequent cultivation of varieties released after 2010 and with growing younger wheat varieties, compared to scenario A, where couples agree that the woman has no role, and scenario C, where the husband is “power-giving”. While the youngest varieties are grown by couples in which the wife is “power-taking” (scenario D), couples in this scenario are found to have tried out new varieties between 2015 and 2021 less frequently. This may be an indication that albeit growing relatively younger varieties, households in disagreement scenario D introduced newer wheat varieties some time ago. Using DNA fingerprinting for wheat variety identification, Hodson et al. (2020) have shown that the adoption of rust-resistant varieties largely occurred between the 2014–15 and 2016–17 growing seasons.

The mean outcomes shown in Table 8 do not control for potential confounding factors. We analyze associations between spousal agreement on the woman’s role and influence in household wheat varietal choice using IPWRA. The results are presented in Table 10. The underlying treatment and outcome models include the same set of additional covariates as in previous models (Table 7, Table S3).

**Table 10:** Associations between agreement on women’s role in wheat varietal choice and outcome variables.

	Household adopted wheat variety released after 2010 (dummy) Based on wife's or husband's interview		Average age of grown wheat varieties (years) Based on wife's or husband's interview		Household has tested new varieties between 2015 and 2021 (dummy) Based on wife's or husband's interview	
	(1)	(2)	(3)	(4)	(5)	(6)
						
**Average Treatment effect on the Treated (ATT)**						
Agreement on wife has role (scenario B) *vs. agreement on wife has no role (scenario A)*	0.09* (0.04)	0.08*(0.04)	−1.31*(0.819)	−1.40* (0.75)	0.06(0.05)	0.02(0.05)
Disagreement, “power-giving” (scenario C) *vs. agreement on wife has no role (scenario A)*	−0.04 (0.06)	0.02 (0.06)	−0.39 (0.96)	−0.58 (0.94)	0.08(0.079)	0.04 (0.06)
Disagreement, “power-taking” (scenario D) *vs. agreement on wife has no role (scenario A)*	−0.18^***^ (0.07)	−0.15^**^(0.092)	−1.98* (1.03)	−1.16 (1.06)	−0.20^***^(0.071)	−0.21^**^ (0.08)
**Potential-outcome means (POmeans)**						
Agreement on wife has no role (scenario A)	0.58^***^ (0.04)	0.58^***^ (0.04)	13.02^***^ (0.69)	12.88^***^ (0. 664)	0.35^***^(0.04)	0.48^***^ (0.04)
	944	944	743^#^	771^#^	944	944

Notes: Treatment variables are based on  (models 1,3,5) or  (models 2, 4,6) interviews. ATT are average treatment effects on the treated. Standard errors in parentheses are robust. ^#^ Differences in sample size are due to farmers identifying a variety which had no match in the variety register, and therefore for which no varietal age could be calculated. * p < 0.10, ^**^ p < 0.05, ^***^ p < 0.001. Additional covariates include characteristics of wife and husband, household and farm characteristics, information access proxies, and location dummies. The full list of covariates is contained in the supplementary materials (Table S3).

The estimation results indicate that spousal agreement on the woman having a role (scenario B) is associated with higher adoption rates of wheat varieties released after 2010 and lower age of varieties, compared to spousal agreement on no role for the woman (scenario A). Joint decision-making with mutually uncontested spousal roles may yield better outcomes due to larger combined exposure to information, as well as spousal discussion and reflection on potential implications of the varietal choice decision. Although conducted in different domains, previous studies have also found positive effects of spousal agreement on a women’s role in household decision-making on using healthcare services (Allendorf, 2007), and on women’s (Ambler et al., 2021), and children’s well-being (Annan et al., 2021).

The results further indicate that the disagreement scenarios (scenarios C and D) are not associated with better outcomes, compared to the scenario of spousal agreement that the woman does not have a role (scenario A). Treatment effects for the “power-giving” scenario (scenario C) are not statistically different from the scenario of agreement on no role for the woman (scenario A). One possible explanation is that some men may be stating what they perceive is expected from them when they say that their wives have a role in decision-making (scenario C). Households in the scenario in which the wife is “taking power” (scenario D) even tend to adopt and test rust-resistant cultivars less frequently. This is in contrast to the findings of Ambler et al. (2021), who report the “power-taking” scenario to be correlated with better well-being outcomes for women in Bangladesh compared to the scenario of spousal agreement on the wife having no involvement, but resembles the findings of Story and Burgard (2012), who found that the disagreement scenarios were also negatively associated with the use of reproductive health care services in Bangladesh. Annan et al. (2021) find that taking power is better for reproductive and child health, but worse for emotional violence. These findings highlight the complex links between the perception and contestation of individual power bargaining.

## 6 Summary and conclusion

Household decisions, including the decision to adopt agricultural technologies, often result from negotiation, contestation and consensus between wife and husband. In other words, the decisions are taken *inside* the household, not *at* the household. This process is shaped by diverging interests, motivations, and objectives, while its results are determined by different levels of individual bargaining power. While an emerging body of literature shows that the increased influence of women in household decisions is associated with positive socioeconomic, health and nutritional outcomes, few studies have analyzed the links between intra-household decision-making and the adoption of agricultural technologies.

In this study, we used a dataset from Ethiopian wheat-producing households in which we captured perceptions of both wife and husband in dual-headed households with regard to their respective roles in the decision-making process on wheat variety choices. In contrast to most studies analyzing the adoption of agricultural technologies, we collected data from wives and husbands separately, to assess the correlates of women’s role in household decisions and its effects on a household’s crop variety choices and turnover. Our findings indicate that women’s ownership of agricultural land and household assets is strongly associated with their active role in household decisions on wheat varietal choice, and with spousal agreement thereon. Women’s engagement in wheat varietal choice, in turn, is associated with a higher likelihood of growing rust-resistant and younger wheat varieties. In terms of spousal agreement on mutual roles in decision-making, we found that spousal agreement on the woman having a role in making crop variety decisions is weakly associated with higher adoption rates of wheat varieties released after 2010 and lower varietal age, compared to spousal agreement that the woman should have no role. The disagreement scenario in which the woman claims to have a role in household decision-making regarding wheat variety choice (and the man claims she has none) is associated with lower adoption rates of rust resistant wheat varieties, and less frequent testing of new wheat varieties in the 2015–21 growing seasons. However, the results are sensitive as to whether the wives' or husbands' interviews are used to construct treatment and outcome variables. In this context, the following implications arise:

The dynamics in intra-household decision-making are likely to influence households’ adoption of agricultural technologies. Obtaining a more nuanced view of intra-household decision-making seems essential to enhance our understanding of the diffusion of agricultural technologies, and may guide actions to accelerate the replacement of varieties by farmers and contribute to increased crop yields and improved farmer livelihoods. Ignoring the dynamics in decision-making equals implicitly assuming that households are unilateral decision-makers (Quisumbing & Maluccio, 2003), a scenario which probably does not hold true considering the degree of spousal disagreement on mutual roles and influence in crop variety choice.

While enhancing women’s bargaining power and spousal agreement thereon is a development objective in itself, it may be a strategy to accelerate crop varietal uptake and turnover to enhance cropping system resilience and productivity. Finally, our results that indicate that women’s ownership of agricultural land is associated with a more active role in decision-making and an acknowledgment of this role by the husband. The promotion of land titles for female farmers or the issuing of joint ownership or usage rights for both spouses may be an avenue to strengthen women’s agency in household decision-making.

## CRediT authorship contribution statement

**Michael Euler:** Writing – original draft, Formal analysis, Data curation, Conceptualization. **Moti Jaleta:** Writing – review & editing, Data curation, Conceptualization. **Hom Gartaula:** Writing – review & editing, Conceptualization.

## Declaration of competing interest

The authors declare that they have no known competing financial interests or personal relationships that could have appeared to influence the work reported in this paper.

## Data Availability

Data will be made available on request.
