# Supplementary material for: Associations between women’s bargaining power and the adoption of rust-resistant wheat varieties in Ethiopia
Source: World Dev. 2024 Jun;178:106567. doi: 10.1016/j.worlddev.2024.106567 (PMC11004725; doi:10.1016/j.worlddev.2024.106567)
Supplement: Supplementary data 1 [file mmc1.docx]

Supplementary Materials.

Table S 1. Associates of women’s role in decisions on wheat varietal choice.

|  | Wife engages in decisions on wheat varietal choice *(dummy)* | |
| --- | --- | --- |
|  | (1) | (2) |
|  | As per female interview | As per male interview |
| Difference between husband’s and wife’s age *(years)* | -1.68e-4 (2.57e-3) | 1.44e-3 (2.42e-3) |
| Difference between husband’s and wife’s education *(years)* | -6.64e-4 (0.00) | -4.11e-3 (4.63e-3) |
| Adult (>15 years) household members *(number)* | 0.01 (0.01) | 0.02^**^ (0.01) |
| Young (<15 years) household members *(number)* | -0.01 (0.01) | 0.01 (0.02) |
| Difference in duration of residence at location between spouses *(years)* | -2.80e-3^*^ (1.43e-3) | -3.79 e-3^***^ (1.34e-3) |
| Difference in size of farm network between spouses *(∆ number of people)* | -2.42e-3^*^ (1.43e-3) | -1.03e-3 (1.23e-3) |
| Difference in size of institutional network between spouses *(∆ number of institutions)* | 2.20e-3 (0.01) | 0.09^***^ (0.01) |
| Cropping area cultivated by household *(ha)* | -0.08^**^ (0.04) | -0.01 (0.03) |
| Share of cropping area under wheat *(% of cropped area)* | -0.08 (0.07) | -0.13^**^ (0.07) |
| Cropping area with use rights by household *(ha)* | 0.01 (0.02) | 0.04^*^ (0.02) |
| Size of land with wife’s or joint use rights is equal to size of land with husband’s use rights (*dummy) ^#^* | -0.05 (0.09) | 0.34^***^ (0.09) |
| Size of land with wife’s or joint use rights is larger than size of land with husband’s use rights *(dummy) ^#^* | 0.27^***^ (0.03) | 0.20^***^ (0.03) |
| Wife’s or joint ownership of non-agricultural items is equal to husband’s ownership of non-agricultural items (*dummy) ^#^* | 0.09^***^ (0.03) | 0.09^***^ (0.03) |
| Wife’s or joint ownership of non-agricultural items is larger than husband’s ownership of non-agricultural items *(dummy) ^#^* | 0.14^**^ (0.06) | 0.02 (0.06) |
| Wife’s or joint ownership of agricultural items is equal to husband’s ownership of agricultural items (*dummy) ^#^* | 0.07^**^ (0.03) | 0.05 (0.03) |
| Wife’s or joint ownership of agricultural items is larger than husband’s ownership of agricultural items *(dummy) ^#^* | 0.05 (0.08) | 0.12 (0.08) |
| Wife owns a cell phone *(dummy)* | -0.10^***^ (0.03) | -0.03 (0.03) |
| Household resides in SNNP region *(dummy)*^##^ | -0.16^***^ (0.06) | -0.01 (0.06) |
| Household resides in Oromia region *(dummy)*^##^ | 0.11^**^ (0.04) | -0.15^***^ (0.05) |
| N | 944 | 944 |
| Pseudo R^2^ | 0.15 | 0.14 |
| Log likelihood | -553.70 | -560.48 |
| chi^2^ | 146.64 | 163.12 |

*Notes: Differences between husband and wife variables are calculated as husband – wife. Results are based on binary logit models. Dependent variable takes on the value 1 if the wife is the main decision maker in household* decisions regarding wheat varietal choice, *or* if *the decision is joint, and 0 otherwise. Standard errors in parentheses are robust. Estimates are marginal effects indicating the change in probabilities of observing the outcome variable as the independent variable increases by one unit. ^*^ p < 0.1, ^**^ p < 0.05, ^***^ p < 0.01^#^ the reference category is lower female or joint ownership of the respective variable in relation to husband’s sole ownership. ^##^ the reference category is households residing in the Amhara region.*

Table S 2. Associates of agreement on women’s role in household crop production decisions*.*

|  | Spousal agreement on female role in wheat varietal choice | | |
| --- | --- | --- | --- |
|  | Agreement on female role | Disagreement, husband assigns greater role to wife | Disagreement, wife assigns herself greater role than husband |
| Difference between husband’s and wife’s age *(years)* | 1.00 | 1.04^**^ | 1.03^*^ |
|  | (0.02) | (0.02) | (0.02) |
| Difference between husband’s and wife’s education *(years)* | 0.98 | 0.99 | 1.01 |
|  | (0.03) | (0.03) | (0.03) |
| Adult (>15 years) household members *(number)* | 1.14^*^ | 1.07 | 0.97 |
|  | (0.08) | (0.08) | (0.08) |
| Young (<15 years) household members *(number)* | 1.01 | 1.03 | 0.87 |
|  | (0.10) | (0.10) | (0.09) |
| Difference in duration of residence at location between spouses *(years)* | 0.97^***^ | 0.98^**^ | 0.98^*^ |
|  | (0.01) | (0.01) | (0.01) |
| Difference in size of farm network between spouses *(∆ number of people)* | 0.99^*^ | 0.99 | 0.98^***^ |
|  | (0.01) | (0.01) | (0.01) |
| Difference in size of institutional network between spouses *(∆ number of institutions)* | 1.45^***^ | 1.61^***^ | 0.89 |
|  | (0.12) | (0.13) | (0.12) |
| Cropping area with use rights by household *(ha)* | 0.56^**^ | 0.97 | 0.66 |
|  | (0.14) | (0.18) | (0.24) |
| Share of cropping area under wheat *(% of cropped area)* | 0.29^***^ | 1.28 | 1.72 |
|  | (0.13) | (0.56) | (0.77) |
| Cropping area with use rights by household *(ha)* | 1.25 | 1.28^*^ | 1.00 |
|  | (0.18) | (0.18) | (0.29) |
| Size of land with wife’s or joint use rights is equal to size of land with husband’s use rights (*dummy) ^#^* | 4.20^**^ | 3.92^***^ | 0.00^***^ |
|  | (2.82) | (1.93) | (0.00) |
| Size of land with wife’s or joint use rights is larger than size of land with husband’s use rights *(dummy) ^#^* | 7.15^***^ | 1.87^***^ | 2.69^***^ |
|  | (1.81) | (0.39) | (0.67) |
| Wife’s or joint ownership of non-agricultural items is equal to husband’s ownership of non-agricultural items (*dummy) ^#^* | 2.07^***^ | 2.07^***^ | 2.24^***^ |
|  | (0.44) | (0.43) | (0.55) |
| Wife’s or joint ownership of non-agricultural items is larger than male spouse ownership of non-agricultural items *(dummy) ^#^* | 2.09^*^ | 0.85 | 2.20^*^ |
|  | (0.85) | (0.39) | (0.91) |
| Wife’s or joint ownership of agricultural items is equal to husband’s ownership of agricultural items (*dummy) ^#^* | 1.71^**^ | 1.01 | 1.08 |
|  | (0.37) | (0.21) | (0.25) |
| Wife’s or joint ownership of agricultural items is larger than husband’s ownership of agricultural items *(dummy) ^#^* | 2.28 | 1.84 | 1.34 |
|  | (1.16) | (0.98) | (0.71) |
| Wife owns a cell phone *(dummy)* | 0.58^**^ | 1.18 | 0.75 |
|  | (0.12) | (0.26) | (0.17) |
| Household resides in SNNP region *(dummy)*^##^ | 0.47^*^ | 1.68 | 1.88 |
|  | (0.18) | (0.58) | (1.29) |
| Household resides in Oromia region *(dummy)*^##^ | 0.88 | 0.72 | 9.18^***^ |
|  | (0.25) | (0.20) | (5.26) |
| Constant | 0.35^*^ | 0.13^***^ | 0.06^***^ |
|  | (0.20) | (0.07) | (0.05) |
| N | 944 | | |
| Pseudo R2 | 0.17 | | |
| Log likelihood | -1063.99 | | |
| Chi2 | 1979.96 | | |

*Notes: Results are based on multinominal logit models. Dependent variable is the level of agreement on the female role in household decisions on wheat varietal choice. Standard errors in parentheses are robust. Estimates indicate the relative risk ratios of observing the respective level of the outcome variable relative to observing the base level (agreement on no female role in wheat varietal choice) as the independent variable increases by one unit. ^*^ p < 0.1, ^**^ p < 0.05, ^***^ p < 0.01. ^#^ The reference category is the wife owning less of the respective variable in relation to the husband. ^##^ The reference region is Amhara.*

Table S 3. Covariates included in the treatment and/or outcome equations of IPWRA models.

|  | N | Mean | Standard deviation | Min | Max |
| --- | --- | --- | --- | --- | --- |
| Age of wife and husband *(average age in years) ^b^* | 944 | 45.73 | 10.19 | 22.5 | 79 |
| Difference in age between husband and wife *(in years) ^a^* | 944 | 9.21 | 6.92 | -35 | 50 |
| Education of wife and husband *(average education in years) ^b^* | 944 | 2.38 | 2.68 | 0 | 14 |
| Difference in education between husband and wife (*in years) ^a^* | 944 | 2.28 | 3.39 | -8 | 13 |
| Difference in years of residence at current location between husband and wife *(in years) ^a^* | 944 | 15.72 | 12.61 | -46 | 72 |
| Size of household farm network *(number of people) ^b^* | 944 | 28.34 | 40.40 | 0 | 489 |
| Wife’s farm network is smaller than husband’s farm network (dummy) *^a^* | 944 | 0.55 | 0.50 | 0 | 1 |
| Wife’s farm network is of equal size than husband’s farm network (dummy) *^a^* | 944 | 0.29 | 0.45 | 0 | 1 |
| Wife’s farm network is larger than husband’s farm network (dummy) *^a^* | 944 | 0.16 | 0.37 | 0 | 1 |
| Institutions husband and wife are members of *(number) ^b^* | 944 | 3.67 | 2.33 | 0 | 12 |
| Wife’s institutional network is smaller than husband’s institutional network (dummy) *^a^* | 944 | 0.46 | 0.50 | 0 | 1 |
| Wife’s institutional network is of equal size than husband’s institutional network (dummy) *^a^* | 944 | 0.51 | 0.50 | 0 | 1 |
| Wife’s institutional network is larger than husband’s institutional network (dummy) | 944 | 0.04 | 0.19 | 0 | 1 |
| Household ownership of mobile phones *(number owned) ^b^* | 944 | 0.83 | 0.89 | 0 | 6 |
| Cropping area cultivated by household *(ha) ^a^ ^b^* | 944 | 0.81 | 0.71 | 0.08 | 8 |
| Cropping area with use rights by household *(share of cropped area) ^a b^* | 944 | 0.84 | 0.94 | 0 | 1 |
| Size of land with wife’s or joint use rights is smaller than size of land with husband’s use rights (*dummy) ^a^* | 944 | 0.30 | 0.46 | 0 | 1 |
| Size of land with wife’s or joint use rights is equal to size of land with husband’s use rights (*dummy) ^a^* | 944 | 0.03 | 0.16 | 0 | 1 |
| Size of land with wife’s or joint use rights is larger than size of land with husband’s use rights (*dummy) ^a^* | 944 | 0.67 | 0.47 | 0 | 1 |
| Share of cropping area under wheat (*share of cropped area*) *^b^* | 944 | 0.46 | 0.24 | 0.04 | 1 |
| Wheat seeds for 2021 season from market - as per female interview *(dummy) ^b^* | 944 | 0.22 | 0.42 | 0 | 1 |
| Wheat seeds for 2021 season from market - as per male interview *(dummy) ^b^* | 944 | 0.22 | 0.42 | 0 | 1 |
| Wheat seeds for 2021 season from agro-dealer - as per female interview *(dummy) ^b^* | 944 | 0.11 | 0.32 | 0 | 1 |
| Wheat seeds for 2021 season from agro-dealer - as per male interview *(dummy) ^b^* | 944 | 0.12 | 0.32 | 0 | 1 |
| Wheat seeds for 2021 from own stocks - as per female interview *(dummy) ^b^* | 944 | 0.50 | 0.50 | 0 | 1 |
| Wheat seeds for 2021 from own stocks - as per male interview *(dummy) ^b^* | 944 | 0.51 | 0.50 | 0 | 1 |
| Wife’s or joint ownership of non-agricultural items is smaller than husband’s ownership of non-agricultural items (*dummy) ^a^* | 944 | 0.43 | 0.50 | 0 | 1 |
| Wife’s or joint ownership of non-agricultural items is equal to husband’s ownership of non-agricultural items (*dummy) ^a^* | 944 | 0.51 | 0.50 | 0 | 1 |
| Wife’s or joint ownership of non-agricultural items is larger than husband’s ownership of non-agricultural items (*dummy) ^a^* | 944 | 0.07 | 0.25 | 0 | 1 |
| Wife’s or joint ownership of agricultural items is smaller than husband’s ownership of agricultural items (*dummy) ^a^* | 944 | 0.36 | 0.48 | 0 | 1 |
| Wife’s or joint ownership of agricultural items is equal to husband’s ownership of agricultural items (*dummy) ^a^* | 944 | 0.59 | 0.49 | 0 | 1 |
| Wife’s or joint ownership of agricultural items is larger than husband’s ownership of agricultural items (*dummy) ^a^* | 944 | 0.05 | 0.21 | 0 | 1 |
| Household resides in SNNP region *(dummy) ^a b^* | 944 | 0.15 | 0.35 | 0 | 1 |
| Household resides in Oromia region *(dummy) ^a b^* | 944 | 0.63 | 0.48 | 0 | 1 |
| Household resides in Amhara region *(dummy) ^a b^* | 944 | 0.23 | 0.42 | 0 | 1 |

*Notes: N is the total number of observations. ^a^ indicates variable is contained in the treatment, ^b^ indicates variable is contained in the outcome equation of IPWRA models. Regional dummies are not contained in the IPWRA outcome models followed by multinominal logit treatment. Covariates recorded through wife (husband) interviews only enter models in which outcome variables are recorded through wife (husband) interview.*

| ** |
| --- |
| Figure S 1. Probability density plot from treatment model of female role in wheat varietal choice, as per female interview.  *Notes: The graph displays the estimated density of the predicted probabilities that a woman not taking part in wheat variety selection decisions is not taking part in wheat variety selection decisions, and that a woman taking part in wheat variety selection decisions is not taking part in wheat variety selection decisions.* |

| ** |
| --- |
| Figure S 2. Probability density from treatment model of female role in wheat varietal choice, as per male interview.  *Notes: The graph displays the estimated density of the predicted probabilities that a woman not taking part in wheat variety selection decisions is not taking part in wheat variety selection decisions, and that a woman taking part in wheat variety selection decisions is not taking part in wheat variety selection decisions.* |
